# Sin3a is essential for the genome integrity and viability of pluripotent cells

**DOI:** 10.1016/j.ydbio.2011.12.019

**Published:** 2012-03-01

**Authors:** Patrick McDonel, Jeroen Demmers, David W.M. Tan, Fiona Watt, Brian D. Hendrich

**Affiliations:** a Wellcome Trust Centre for Stem Cell Research and MRC Centre for Stem Cell Biology and Regenerative Medicine, University of Cambridge, Tennis Court Road, Cambridge CB2 1QR, UK; b Institute for Stem Cell Research and MRC Centre for Regenerative Medicine, University of Edinburgh, Edinburgh EH9 3JQ, UK; c Proteomics Center, Erasmus University Medical Centre, Postbus 2040, 3000 CA Rotterdam, The Netherlands; d Department of Biochemistry, University of Cambridge, Tennis Court Road, Cambridge CB2 1GA, UK; e Cancer Research UK Cambridge Research Institute, Li Ka Shing Centre, Robinson Way, Cambridge CB2 0RE, UK

**Keywords:** Sin3a, ICM, Apoptosis, Embryonic stem cells, Cell cycle, DNA damage

## Abstract

The Sin3a/HDAC co-repressor complex is a critical regulator of transcription networks that govern cell cycle control and apoptosis throughout development. Previous studies have identified Sin3a as essential for embryonic development around the time of implantation, during which the epiblast cell cycle is uniquely structured to achieve very rapid divisions with little tolerance of DNA damage. This study investigates the specific requirement for Sin3a in the early mouse embryo and shows that embryos lacking Sin3a suffer unresolved DNA damage and acute p53-independent apoptosis specifically in the E3.5–4.5 epiblast. Surprisingly, Myc and E2F targets in *Sin3a*-null ICMs are downregulated, suggesting a central but non-canonical role for Sin3a in regulating the pluripotent embryonic cell cycle. ES cells deleted for Sin3a mount a DNA damage response indicative of unresolved double-strand breaks, profoundly arrest at G2, and undergo apoptosis. These results indicate that Sin3a protects the genomic integrity of pluripotent embryonic cells and governs their unusual cell cycle.

## Highlights

► Sin3a is required for proliferation and survival of pluripotent embryonic cells. ► Sin3a-/- mouse ICM cells are eliminated by p53-independent apoptosis. ► Sin3a is a critical regulator of the unusual pluripotent embryonic cell cycle. ► Sin3a prevents or repairs DSBs that arise in ES cells, perhaps during replication. ► The Sin3a interactome provides insights into function Sin3a in ES cells.

## Introduction

Robust mechanisms are required to protect the genomic integrity of pluripotent embryonic cells that give rise to the entire organism and future generations through the germ line. Consequently, embryonic stem (ES) cells and, by inference, cells of the inner cell mass (ICM) have considerably lower mutation frequencies than somatic cells (Hong et al., 2007) and are highly sensitive to DNA damage and other genotoxic stresses (de Waard et al., 2003; Roos et al., 2007; Van Sloun et al., 1999).

The intolerance of ES cells toward DNA damage is facilitated by elaborate mechanisms compromising the G1 damage checkpoint and abating the opportunity for repair, routing damaged cells to the more stringent, intact G2/M checkpoint and ultimately apoptosis. In self-renewing ES cells, checkpoint proteins such as p53 (encoded by the *Trp53* gene) are sequestered almost entirely in the cytosol, thus preventing effective regulation of their nuclear targets in response to DNA damage (Aladjem et al., 1998; Hong and Stambrook, 2004; Lin et al., 2005; Solozobova et al., 2009). Indeed, ES cells deleted for *Trp53* readily undergo apoptosis in response to DNA damage with kinetics similar to wild-type cells (Aladjem et al., 1998; Lin et al., 2005; Solozobova et al., 2009), despite a failure to induce canonical somatic targets of p53.

As with the response to DNA damage, models of cell cycle control in the epiblast are largely inferred from studies of mouse ES cells (for reviews see (Orford and Scadden, 2008; White and Dalton, 2005)). The extremely rapid divisions of mouse ES cells are accomplished by the effective elimination of the early G1 phase. Unlike in somatic cells, constitutively high expression of Cyclin E1 stimulates Cdk2 activity throughout the entire ES cell cycle, thereby maintaining Rb in a hyperphosporylated, inactive state and bypassing both the restriction point (R) and the need for mitogen stimulation of Cyclin D-Cdk4/6 activity (Burdon et al., 2002; Savatier et al., 1994; White et al., 2005). This constitutive inactivation of Rb results in continual activation by E2f1 of its targets, including *Cyclin E1* (MGI: *Ccne1)* and the replication machinery, thus rapidly driving ES cells from mitosis into S phase regardless of any damage or stress that might have occurred.

A key regulator of the Myc, E2F, and p53 transcription networks that govern cell cycle control and apoptosis throughout development is the Sin3a co-repressor complex (McDonel et al., 2009; Silverstein and Ekwall, 2005). This conserved complex is scaffolded by the Sin3a protein, which binds class I histone deacetylases (HDACs) Hdac1 and Hdac2 (Laherty et al., 1997) and a diverse array of sequence-specific repressors via its paired amphipathic helices (Le Guezennec et al., 2006; Sahu et al., 2008), thus recruiting HDAC activity to target promoters.

Sin3a function is essential for the growth and viability of mouse embryonic fibroblasts (MEFs). Deletion of *Sin3a* in MEFs results in a profound growth defect, significant G2/M accumulation, and increased apoptosis in conjunction with de-repression of hundreds of Myc, E2f1, E2f4, and p53 targets that control cell cycle progression, DNA replication and repair, and cell death (Dannenberg et al., 2005). Interestingly, while the induction of *p21* ^*Cip*^ (MGI: *Cdkn1a*) in response to *Sin3a* deletion requires p53, the growth arrest, replication defects, and apoptosis were not alleviated by either ablation or functional inactivation of p53. Furthermore, genes involved in both non-homologous end-joining (NHEJ) and homologous recombination (HR) repair pathways were aberrantly upregulated in MEFs lacking *Sin3a* (Dannenberg et al., 2005), suggesting a novel role in balancing the relative activities of these two double-strand break (DSB) repair strategies in addition to its reported chromatin-modifying functions during NHEJ (Jazayeri et al., 2004) and DNA replication (Aparicio et al., 2004) in yeast.

Genetic studies have shown that *Sin3a* is required for early mouse embryonic development between E3.5 and gastrulation at E6.5 (Cowley et al., 2005; Dannenberg et al., 2005). Similarly, knock-down of Sin3a by siRNAs resulted in severely impaired proliferation in ES cells (Fazzio et al., 2008). However, as outlined above, many of the genes overexpressed in *Sin3a* ^−/−^ MEFs that led to growth defects and apoptosis are already highly expressed in early embryonic cells, and thus their de-repression in the ICM or ES cells is not necessarily expected to have such adverse effects. Therefore, the mechanisms underlying this embryonic requirement for Sin3a are not at all clear.

To better understand the requirement for Sin3a/HDAC complexes in early mammalian embryogenesis, we examined in detail embryos and ES cells deleted for *Sin3a*. We found that *Sin3a* is absolutely required for proliferation and survival of cells in the ICM as embryos implant, but cells in the trophectoderm lineage appeared largely normal in *Sin3a* ^−/−^ embryos. We identify a number of proteins interacting with Sin3a in ES cells, further implicating the complex in cell cycle control and the DNA damage response. We propose that Sin3a is essential to maintain both the unusual cell cycle of pluripotent embryonic cells and their genomic integrity.

## Materials and methods

### Mice and embryos

*Sin3a* ^*Flox/+*^ ES cells were a kind gift of Gregory David (New York). ES cells were injected into C57Bl/6 host blastocysts to generate chimeric mice using standard methods (Hogan et al., 1994). Resulting *Sin3a* ^*Flox/+*^ mice were then either intercrossed to create a homozygous *Sin3a* ^*Flox/Flox*^ line or crossed to mice expressing a Cre transgene under the control of the *Sox2* promoter (Hayashi et al., 2002), kindly provided by Jennifer Nichols (Cambridge). Maternal contribution of the Cre protein to the zygote resulted in recombination between the LoxP sites and creation of *Sin3a* ^+/−^ mice, which were maintained as a heterozygote stock by outcrossing to C57Bl/6 mice. Mice were genotyped in a duplex PCR reaction using the following primers: S3AP2, CAGATCCTATTCCAGGTGTCAAAG; S3AP6, GGGGGAATGCTGTGTTTTAGGTATG; and S3AP12, CAAGTTCATCAGATTCCCAC. Genotyping of embryos was performed using nested PCR consisting of 25 cycles with standard genotyping primers, followed by 20 cycles with the following primers: S3AP9, CATCCTTCCCAGCCTTCAT; S3AP10, TACAAAGCCAGCCCTGAGAC; and S3AP11, CAAGATGGCCTTGAACTTTTGG.

ES cell derivation, ICM outgrowths in ERK inhibitor, and induction of diapause were all performed as described (Buehr and Smith, 2003; Nichols et al., 2009).

All mice were housed under standard conditions. All procedures were covered by a license granted by the UK Home Office and were approved by institutional ethics committees.

### Immunofluorescence

Embryos were fixed in 2.5% paraformaldehyde, permeabilised in 0.25% Triton X-100 in PBS, and blocked in PBS containing either 3% donkey serum, 0.1% BSA, and 0.01% Tween-20 or 10% fetal calf serum and 0.1% Triton X-100. The primary antibodies were applied in blocking solution at 4 °C overnight, and secondary antibodies were applied for an hour at room temperature. Primary antibodies used were the following: anti-Sin3a (1/200, sc-768, Santa Cruz Biotechnology), anti-Oct4 (1/100, sc-5279 and sc-8628, Santa Cruz Biotechnology), anti-Nanog (1/250, ab21603, Abcam and RCAB0002P-F, Cosmo Bio Co), anti-Cdx2 (1/200, Cdx2-88, BioGenX), anti-Eomes (1/200, ab23345, Abcam), anti-Gata4 (1/200, sc-1237, Santa Cruz Biotechnology), anti-Mi-2ß (1/200, 39289, Active Motif), anti-activated Caspase 3 (1/500, AF835, R&D Systems), and anti-phospho-H2A.X (S139) (1/500, 05-636, Millipore). For the phoshpo-H2A.X (γH2AX) quantitation, mean staining intensity values obtained from confocal images were measured for DAPI-positive nuclei using Volocity software (Perkin Elmer) for three wild-type embryos, eight heterozygous embryos, and six null embryos. Cells were scored as being ICM or TE based upon Oct4 expression levels. This resulted in 78, 184, and 96 wild-type, heterozygous, or null ICM cells respectively, and 118, 250, and 216 wild-type, heterozygous and null TE cells, respectively. Mean intensity values of anti-H2AX staining were used to build box plots (http://www.physics.csbsju.edu/stats/display.distribution.html). P-values were calculated using a single tailed Mann–Whitney test (http://faculty.vassar.edu/lowry/utest.html).

### Single-ICM gene expression analyses

Individual ICMs from *Sin3a* ^+/−^ intercrosses were dissected away from trophectoderms by immunosurgery (Nagy et al., 2006) at 3.5 dpc and placed into either first-strand buffer [1 × Superscript III buffer (Invitrogen), 0.5% NP-40 (Pierce), 10 mM dNTP mixture (Roche), 3.4 nM MO_4_dT_30_ primer (AAGCAGTGGTATCAACGCAGAGTGGCCATTACGGCCGT AC-(dT)_30_) (Osawa et al., 2005), 1 mM DTT (Invitrogen), 5 U SuperRNaseIN (Ambion), 7.5 U PrimeRNase inhibitor (Eppendorf)] or miRNA lysis buffer [1 mg/mL BSA (NEB), 0.5% NP-40 (Pierce)], snap-frozen on dry ice, and stored at – 80 °C. Trophectoderms from each embryo were lysed in PCR lysis buffer [1 × ThermoPol buffer (NEB), 0.45% Igepal CA-630 (Sigma), 0.45% Tween 20 (Sigma), 200 μg/mL Proteinase K (Roche)] at 55 °C for 2 h followed by inactivation for 15 min at 95 °C and genotyped for *Sin3a* by duplex PCR as described above.

For cDNA synthesis, ICMs frozen in first-strand buffer were lysed at 65 °C for 5 min, then diluted 1:10 in 4.5 μL first-strand buffer and re-heated to 65 °C for 5 min. Primer was allowed to anneal at 45 °C for 2 min before addition of 0.5 μL of Superscript III and incubation at 45 °C for 15 min. The reaction was inactivated at 65 °C for 10 min. Unannealed primer was digested by the addition of 4 U Exonuclease I (Thermo Fisher Scientific) in the presence of 6.7 mM MgCl_2_ in 1.0 μL and incubated at 37 °C for 30 min before inactivation at 80 °C for 25 min. Removal of the RNA template and polyadenylation of the cDNA was carried out concurrently by the addition of 5 U RNaseH (Invitrogen), 2.6 μL of 5× terminal deoxynucleotidyltransferase buffer (Promega) supplemented with 1.5 mM dATP (Roche) and 30 U terminal deoxynucleotidyltransferase (TdT) (Promega), incubating for 15 min at 37 °C before inactivating at 65 °C for 10 min. 4 μL of polyadenylated cDNA was used as template for PCR amplification in 1× ExTaq buffer (TaKaRa), 0.65 mM dNTP (Roche), 8.25 μM MO_4_dT_30_ primer, 5 U ExTaq (TaKaRa) by incubating at 94 °C for 1 min, 50 °C for 2 min, and 72 °C for 2 min to allow second-strand synthesis. Subsequently, 35 cycles of amplification were performed by incubating at 94 °C for 30 s, 60 °C for 30 s, and 72 °C for 2 min. The first round of amplification was performed in triplicate, after which the amplified cDNA was pooled. A second round of amplification was performed in duplicate as above by using 2 μL of the pooled amplified cDNA as template. The amplified cDNA was again pooled before purification with the QIAquick PCR purification kit (Qiagen), and the eluate diluted 1:50 in water for subsequent qPCR using an Applied Biosystems StepOne Plus PCR system with Roche SYBR Green Master reagents and primers listed in Suppl. Table 1.

Reverse transcription and qPCR analysis of miRNAs were conducted exactly as described (Tang et al., 2006b), using only the gene-specific forward primers listed in Suppl. Table 1 for the cDNA amplification step.

### ES cells

*Sin3a^Flox/−^* ES cells were derived from mixed C57/129 blastocysts and cultured in ES cell medium supplemented with 10% fetal bovine serum and recombinant human LIF on gelatin-coated flasks, as described (Smith, 1991). Stable integration of a plasmid expressing Cre-ER^T2^-IRES-Puro from a CAG promoter was achieved by electroporating 5 × 10^7^ *Sin3a^Flox/−^* ES cells with 80 μg linearised vector, plating of serial dilutions, and selection with 1 μg/mL puromycin for 6 days until colonies were clearly visible. Clones were picked into individual gelatin-coated wells and subsequently expanded under puromycin selection. In transfected lines, Cre was translocated into the nucleus by the addition of 400 nM 4-hydroxytamoxifen. Apoptosis was detected in unfixed ES cells using Alexa Fluor 488 Annexin V (Invitrogen) following the manufacturer's instructions, except for including 1 μg/mL DAPI as a live/dead discriminator in place of propidium iodide. Western blots were performed on total ES cell lysates prepared in Laemmli buffer added directly to culture dishes. The following antibodies were used: anti-Sin3a (1/500, sc-994, Santa Cruz), anti-phospho-histone H2A.X (Ser139) (1/2000, 05-636, Millipore), anti-phospho-SMC1 (Ser966) (1/2000, generous gift of John Rouse, University of Dundee), anti-phospho-53BP1 (Ser166) (1/320, S651B, generous gift of John Rouse, University of Dundee) and anti-α-tubulin (1/1000, sc-53646, Santa Cruz).

### Cell cycle analysis by flow cytometry

At the indicated time points, approximately 1–2 × 10^5^ ES cells were trypsinised, washed in PBS, and fixed dropwise into ice-cold 70% ethanol. Fixed cells were washed twice with wash buffer (PBS plus 2.5% Fetal Bovine Serum), then permeabilised in wash buffer containing 0.25% Triton X-100 for 15 min on ice. Permeabilised cells were then incubated for 3 h at room temperature in wash buffer containing 100 μg/mL mouse anti-phospho-histone H3 (S10) antibody (Sigma), washed twice, and stained with an Alexa Fluor 488-conjugated anti-mouse secondary antibody (Invitrogen) for 30 min. After two more washes, cells were stained with 50 μg/mL propidium iodide (PI) and fluorescence was measured using a FACSCalibur flow cytometer (BD Biosciences). At least 10,000 gated events were recorded for each sample. Positive/negative scoring for phospho-histone H3 was determined by comparison to control cells stained with PI alone and applied uniformly across all samples. P-values were calculated using Fisher's exact test.

### Sin3a-3xFLAG expression and immunoprecipitation

Sin3a cDNA was synthesised from ES cell total RNA using reverse transcription and cloned in-frame into the C-terminal 3x-FLAG expression vector pCAG-A3XF-iH. Following transfection into E14tg2a ES cells using Lipofectamine 2000 (Invitrogen) and 6 days of selection with 100 μg/mL hygromycin, resistant colonies expressing either Sin3a-3xFLAG or 3xFLAG alone (empty vector) were expanded and assayed for endogenous Sin3a and Sin3a-3xFLAG expression levels by western blot. Two independent Sin3a-3xFLAG clones and two vector-only clones were chosen and expanded for preparation of nuclear extracts. Briefly, cells were harvested by trypsinisation and rinsed twice in ice-cold PBS, then once in ice-cold lysis buffer (10 mM HEPES-KOH pH 7.6, 10 mM KCl, 1.5 mM MgCl_2_, 0.5 mM PMSF, 0.5 mM DTT). Cells were suspended in lysis buffer for 10 min on ice, then gently lysed using a glass dounce homogeniser. Nuclei were pelleted by centrifugation at 3300 *g* and resuspended in low-salt nuclear extract buffer (25 mM HEPES-KOH pH 7.6, 20 mM KCl, 1.5 mM MgCl_2_, 10% glycerol, 0.5 mM PMSF, 0.5 mM DTT) and then lysed by adding KCl to 450 mM and Igepal CA-630 to 0.15% and rotating at 4 °C for 1 h. Insoluble material was pelleted by centrifugation for 30 min at 20,000 *g*, and the supernatant was aliquoted and flash-frozen in liquid nitrogen.

For immunoprecipitations, 13–15 mg nuclear extract was diluted in IP buffer [25 mM HEPES-KOH pH 7.6, 140 mM KCl, 10% glycerol, 0.1% Igepal CA-630, 0.5 mM EDTA, 0.5 mM DTT, 1X Protease Inhibitor Cocktail III (Calbiochem)], pre-cleared against protein G sepharose, and then rocked for 4 hours at 4 °C with 75 μL (packed) anti-FLAG M2 resin (Sigma). Following extensive washes in IP buffer containing 250 mM KCl, Sin3a complexes were eluted 3 times in IP buffer containing 200 μg/mL 3× FLAG peptide for 30 min. Eluates were pooled and precipitated with trichloroacetic acid. The pellets were washed in acetone and solubilised in Laemmli buffer for SDS-PAGE on separate 4–12% Bis–Tris Novex (Invitrogen) polyacrylamide gels for each sample.

### Mass spectrometric analysis

1D SDS-PAGE gel lanes were cut into 2 mm slices using an automatic gel slicer and subjected to in-gel reduction with DTT alkylation with iodoacetamide, and digestion with trypsin (Promega, sequencing grade), essentially as described (Wilm et al., 1996). Nanoflow LC-MS/MS was performed on an 1100 series capillary LC system (Agilent Technologies) coupled to an LTQ-Orbitrap mass spectrometer (Thermo) operating in positive mode and equipped with a nanospray source. Peptide mixtures were trapped on a ReproSil C18 reversed phase column (Dr Maisch GmbH; column dimensions 1.5 cm × 100 μm, packed in-house) at a flow rate of 8 μL/min. Peptide separation was performed on ReproSil C18 reversed phase column (Dr Maisch GmbH; column dimensions 15 cm × 50 μm, packed in-house) using a linear gradient from 0% to 80% B (A = 0.1% formic acid; B = 80% (v/v) acetonitrile, 0.1% formic acid) over 70 min at a constant flow rate of 200 nL/min using a splitter. The column eluent was directly sprayed into the ESI source of the mass spectrometer. Mass spectra were acquired in continuum mode; fragmentation of the peptides was performed in data-dependent mode. Peak lists were automatically created from raw data files using the Mascot Distiller software (version 2.3; MatrixScience). The Mascot search algorithm (version 2.2, MatrixScience) was used for searching against the SwissProt FASTA protein sequence database (version SwissProt_56.4, taxonomy: *Mus musculus*). The peptide tolerance was set to 10 ppm and the fragment ion tolerance was set to 0.8 Da. A maximum number of 2 missed cleavages by trypsin were allowed and carbamidomethylated cysteine and oxidised methionine were set as fixed and variable modifications, respectively. The Mascot score cut-off value for a positive protein hit was set to 65. Individual peptide MS/MS spectra with Mascot scores below 40 were checked manually and either interpreted as valid identifications or discarded. Typical contaminants, also present in immunopurifications using beads coated with pre-immune serum or antibodies directed against irrelevant proteins, were omitted from the table.

## Results

### Sin3a is required for epiblast formation

**Fig. 1:**
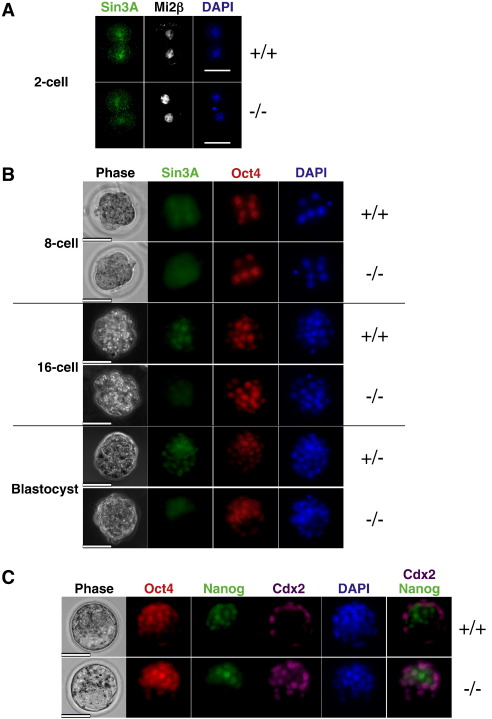
Sin3a expression in preimplantation development. A. Maternal Sin3A protein (green) can be detected as nuclear signal in wild-type and *Sin3a*^−/−^ 2-cell embryos. Mi2β expression is shown in white and DAPI staining in blue. B. Staining of 8- and 16-cell embryos and early blastocysts with an anti-Sin3a antibody (green) shows nuclear signal in wild-type and heterozygotes, but loss of nuclear staining in *Sin3a*^−/−^ embryos from the 8-cell stage onwards. Staining for Oct4 (red) and DAPI (blue) is also indicated. C. Staining wild-type and *Sin3a*^−/−^ blastocysts for the ICM markers Oct4 (red) and Nanog (green), for the trophectoderm marker Cdx2 (pink), and for DAPI (blue). A panel showing both Nanog and Cdx2 staining is shown to highlight segregation of the ICM and TE lineages. For all embryos the genotypes are shown to the right. Scale bar = 50 μm.

To investigate the function of Sin3a in pluripotent cells, we first investigated the role of Sin3a in pre- and peri-implantation development in mice. Nuclear Sin3a protein is visible in both wild-type and *Sin3a* ^−/−^ 2-cell embryos, indicating that Sin3a protein is deposited in the egg and that this maternal protein persists through the first few cleavage divisions (Fig. 1A). By the 8-cell stage nuclear Sin3a is less apparent in mutant embryos than in wild-type littermates, and by the 16-cell stage the maternal protein has largely been depleted and null embryos show no nuclear Sin3a protein (Fig. 1B). At the blastocyst stage Sin3a is expressed ubiquitously in wild-type and heterozygous embryos (Fig. 1B). Despite lacking any detectable Sin3a protein, null blastocysts are able to cavitate and, as judged by patterns of antibody staining for the Oct4, Nanog, and Cdx2 proteins, are able to execute the first lineage decision to segregate inner cell mass (ICM) and trophectoderm (Fig. 1C). Indeed, Sin3a blastocysts were recovered from heterozygote intercrosses at near Mendelian ratios (Table 1 ). As maternal Sin3a is largely depleted by the 8-cell stage, these results indicate that Sin3a does not have an essential role in cellular proliferation or early cell fate specification in late preimplantation stage embryos.

**Fig. 2:**
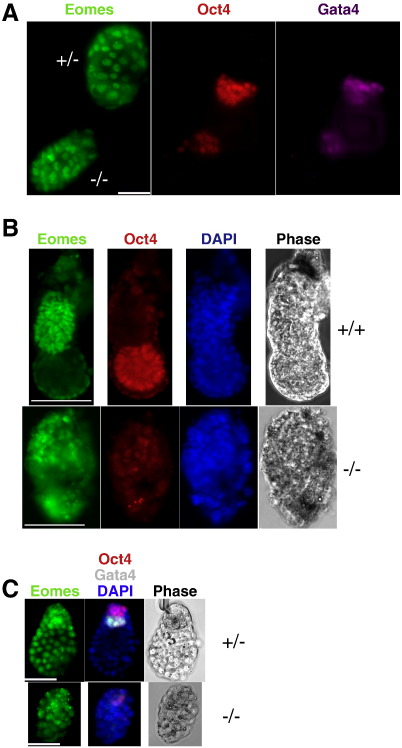
Loss of ICM cells in peri-implantation *Sin3a*-null embryos. A. Heterozygote and null 4.5 dpc embryos stained for Eomes (green), Oct4 (red) and Gata4 (purple). Scale bar = 50 μm. B. 5.5 dpc embryos of indicated genotypes stained for Eomes (green), Oct4 (red) and DAPI (blue). Scale bar = 100 μm. C. *Sin3a*^+/−^ and *Sin3a*^−/−^ embryos recovered after two days of diapause stained for Eomes (green), in the left hand panel, and Oct4 (red), Gata4 (white) and DAPI (blue) in the middle panel. Phase image of the embryos is shown in the right hand panel. Genotypes are indicated to the right. Scale bar = 50 μm.

**Fig. 3:**
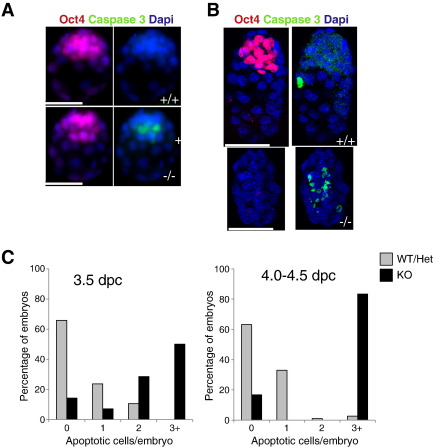
ICM cells are lost by apoptosis in *Sin3*a-null embryos. Examples of embryos recovered at 3.5 dpc (A) and 4.5 dpc (B) stained for Oct4 (red), activated Caspase-3 (green) or DAPI (blue). Quantitation of caspase positive staining in embryos recovered at 3.5 dpc (C) and 4.5 dpc (D). The graphs are derived from 38 wild-type or heterozygote embryos and 18 null embryos at 3.5 dpc (C), and 44 wild-type or heterozygotes and eight null 4.5 dpc embryos (D). Scale bar = 50 μm.

Despite the apparently normal preimplantation development, implanting *Sin3a* ^−/−^ embryos were recovered at sub-Mendelian ratios, and those recovered were obviously smaller than their wild-type and heterozygous littermates, with the Oct4- and Gata4-expressing cell populations appearing to be particularly affected (Table 1, Fig. 2A, and Fig. 3A right panel). Nevertheless, most embryos were able to implant, indicating that trophectoderm cells do not require Sin3a to form an attachment to the uterus, consistent with a previous report that seemingly empty implantation sites could be identified at 6.5 dpc (Cowley et al., 2005). By 5.5 dpc few mutant embryos were recovered, but those identified appeared to have completely lost their embryonic compartment, as judged by Oct4 and Gata4 staining, and consisted entirely of Eomes-expressing trophectoderm (Table 1; Fig. 2B). Therefore, we conclude that Sin3a is absolutely required for the viability and/or proliferation of epiblast cells in peri-implantation embryos.

We next wished to assess whether the specific loss of epiblast cells in Sin3a mutant embryos was an intrinsic property of the embryo or was induced externally during implantation. To distinguish these possibilities, we first examined the status of wild-type and mutant embryos in which the preimplantation period was extended by inducing diapause (Buehr and Smith, 2003). Strikingly, subjecting *Sin3a* ^−/−^ blastocysts to diapause for two days resulted in complete loss of the ICM (Fig. 2C), whereas wild-type (not shown) and heterozygous blastocysts maintained their characteristic ICM morphology. To remove blastocysts from the uterine environment completely, blastocysts were flushed early on the fourth day and allowed to outgrow in standard ES cell serum and LIF conditions. Consistent with a previous report (Cowley et al., 2005), we found that *Sin3a*-null embryos were able to attach to the substrate but completely failed to outgrow (data not shown).

Failure of blastocyst outgrowth could be due either to a general proliferation defect or to a specific failure of the trophectoderm cells. To assess the proliferation potential of pluripotent cells lacking Sin3a, embryos were collected at the 8-cell stage and incubated in the presence of the ERK inhibitor PD0325901 until the expanded blastocyst stage, as described (Nichols et al., 2009). This prevented formation of hypoblast in the resulting blastocysts, allowing us to isolate pure epiblasts after immunosurgery. Outgrowth of isolated wild-type or heterozygous epiblasts in 2i media (Nichols et al., 2009) resulted in derivation of embryonic stem cell lines in all cases, but *Sin3a* ^−/−^ epiblasts failed to outgrow (data not shown). Therefore, we conclude that Sin3a is required for the expansion of pluripotent cell populations both *in vivo* and *ex vivo*.

### Apoptosis specifically eliminates the epiblasts from embryos lacking *Sin3a*

Our data thus far indicated a specific loss of the *Sin3a*-null pluripotent cell population during implantation, but little if any effect upon trophectoderm cells. Sin3a activity has been shown to prevent p53-independent apoptosis in murine embryonic fibroblasts (MEFs) (Cowley et al., 2005; Dannenberg et al., 2005). To determine whether ICM cells of *Sin3a* ^−/−^ embryos were dying via apoptosis, pre- and peri-implantation stage embryos were stained for activated Caspase-3, a marker of apoptotic cells (Nicholson, 1999). Apoptosis occurs at a very low rate in the ICM of wild-type blastocysts during normal development (Jang et al., 2005). Consistent with this, we saw that the vast majority of wild-type or *Sin3a* ^+/−^ blastocysts had either zero or one Caspase 3-positive cell (Fig. 3). By contrast, half of the *Sin3a* ^−/−^ blastocysts had three or more Caspase 3-positive cells, usually located in the ICM (Figs. 3A left panel, B). One day later almost all wild-type or heterozygous embryos had zero or one Caspase 3-positive cell, whereas over 80% of *Sin3a* ^−/−^ embryos showed three or more Caspase 3-positive cells (Figs. 3A right panel, B). Those embryos showing multiple apoptotic cells also showed little or no Oct4 staining, consistent with death of ICM cells in the absence of Sin3a (Fig. 3B). Therefore, we conclude that Sin3a is specifically required for survival of pluripotent cells in implanting blastocysts, but it is not required for development of the trophectoderm until at least 5.5 dpc.

### Apoptosis in *Sin3*^−/−^ ICMs is independent of p53 activity

**Fig. 4:**
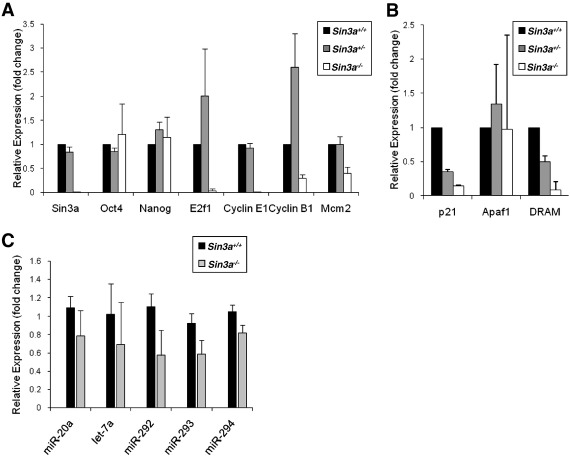
Disruption of cell cycle control gene expression in *Sin3a^−/−^* ICMs. A and B. The mean expression level of each indicated gene normalised to an internal standard (*Gapdh*) is plotted on the Y-axis as fold-change relative to the mean value for all wild-type samples (defined as 1.0). Black bars are the relative values for wild-type, grey bars for *Sin3a*^+/−^, and white bars for *Sin3a*^−/−^. Error bars represent the standard deviation from the mean among biological replicates. C. Mean relative expression of each indicated miRNA in *Sin3a*^+/+^ (black) and *Sin3a*^−/−^ (grey), normalised to *miR-16*, is plotted on the Y-axis as fold-change relative to its mean value in an arbitrarily chosen *Sin3a*^+/+^ sample processed in parallel. Error bars represent standard deviation from the mean among biological replicates.

We next addressed whether the growth defect and apoptosis observed in *Sin3a* ^−/−^ ICMs were the result of p53 activation of arrest and cell death pathways. We found that although E3.5 ICM cells deleted for *Sin3a* were destined for apoptosis, they did not show induction of the p53 targets *p21* (cell cycle arrest), *Apaf-1* [apoptosis, (Moroni et al., 2001)], or *Dram* [autophagy and apoptosis, (Crighton et al., 2006)] (Fig. 4B). By contrast, levels of both *p21* and *Dram* transcripts showed a dose-dependent reduction in *Sin3a* heterozygous and null ICMs, possibly indicating a previously unrecognised relationship between Sin3a and these p53 targets in early embryonic cells. Our observations are consistent with a previous report that MEFs deleted for *Sin3a* undergo p53-independent apoptosis (Cowley et al., 2005) and suggest that the cell death induced in pluripotent cells lacking Sin3a is exerted independently of p53 activity.

### *Sin3a*^−/−^ ICMs fail to express key cell cycle promoting factors

To investigate the underlying causes of the growth and apoptosis defects observed in *Sin3a* ^−/−^ embryos, we compared the transcript levels of key cell cycle regulatory genes in wild-type, heterozygous, and null ICMs at E3.5, the stage at which these phenotypes initially emerged. We found that as early as E3.5 transcripts for key cell cycle regulatory proteins were either not expressed or expressed at substantially reduced levels in ICMs deleted for *Sin3a* as compared with their wild-type or heterozygous counterparts (Fig. 4A). Most notably, whereas *Sin3a* ^−/−^ ICMs express normal levels of *Oct4* and *Nanog*, they expressed nearly undetectable levels of *E2f1* and one of its key targets, *Ccne1* (*Cyclin E1*), both of which are critical for driving ES cells rapidly through G1(Fujii-Yamamoto et al., 2005; Stead et al., 2002). *Sin3a*-null ICM cells also expressed less than half the wild-type levels of the replication licensing factor *Mcm2* and the principal mitotic cyclin *Ccnb1* (*Cyclin B1*). The absence of transcripts for these key cell cycle proteins makes it very unlikely that the cells would be able to progress through DNA replication and mitosis.

### *Sin3a*^−/−^ cell cycle misregulation and cell death phenotypes are not caused by mis-expression of ESCC or Myc-regulated miRNAs

One potential explanation for decreased *E2f1* levels and p53-independent cell cycle disruption (and possibly the resulting cell death) comes from recent studies on miRNA control of the cell cycle, particularly in ES cells. Artificially high levels of c-Myc expression were shown in human B cells to induce the *miR-20* family of miRNAs as part of a feedback loop, resulting in a block in the translation of *E2F1* transcripts (Zhang and Pugh, 2011). This effectively mimics the E2F-inhibitory function of Rb and is expected to prevent expression of E2F targets such as the G1 cyclins and replication factors such as *Mcm2*. Moreover, so-called "ES Cell Cycle Control" (or ESCC) miRNAs in the *miR-290* family stimulate ES cell proliferation by indirectly activating c-Myc and N-Myc, and thus by extension Myc targets, while also blocking p21 translation and inhibiting the Myc inhibitor miRNA *let-7* (Gilchrist et al., 2008, 2010; Kumar et al., 2007). We therefore measured the levels of miRNAs in *Sin3a* ^−/−^ ICMs as compared with wild-type littermate ICMs (Fig. 4C). Our analysis of miRNAs in *Sin3a*-null ICMs revealed no induction of *miR17-5p* nor increased expression of *miR-20a* or *miR-290* family miRNAs, thereby demonstrating that the apoptosis and repression of Myc/E2F targets we observe in Sin3a null ICMs are not due to misregulation of these miRNAs.

### Sin3a protects against DNA double-strand breaks and cell cycle arrest in pluripotent cells

**Fig. 5:**
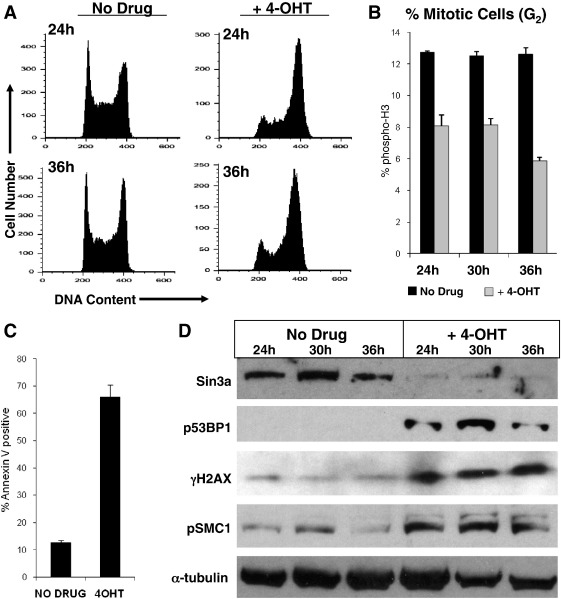
Sin3a is required for cell cycle progression and genome maintenance in ES cells. *Sin3a*^*Flox/–*^*:CreER*^*T2*^ ES cells were either untreated (No Drug) or treated with 400 nM 4-hydroxytamoxifen (+ 4-OHT) and harvested at the indicated times after tamoxifen addition. A. Cell cycle distribution of *Sin3a*^*Flox/–*^*:CreER*^*T2*^ ES cells with and without 4-OHT treatment. DNA content is indicated by propidium iodide staining (X-axis), where 2C is the diploid DNA content in G1 and 4C is the replicated diploid DNA content in G2. B. Mean % of tamoxifen-treated (grey) and untreated (black) *Sin3a*^*Flox/–*^*:CreER*^*T2*^ ES cells with a G2 (4 C) DNA content that are positive for phospho-histone H3 (S10), indicating mitotic entry. Error bars represent standard deviation from the mean among independent replicates, and p < 0.0001 according to Fisher's exact test. C. Mean % of live, unfixed *Sin3a*^*Flox/–*^*:CreER*^*T2*^ ES cells staining positive for Annexin V, indicating apoptosis, after 36 h of tamoxifen (+ 4-OHT) or mock (No Drug) treatment. D. Western blots of total lysates prepared from *Sin3a*^*Flox/–*^*:CreER*^*T2*^ ES cells at the indicated times after tamoxifen addition. Protein names beginning with ‘p’ indicate the antibody specifically recognises the phosphorylated form of the protein.

In an effort to understand why pluripotent cells lacking Sin3a undergo apoptosis, we created an ES cell line in which *Sin3a* could be deleted conditionally. Multiple independent ES cell lines were derived from mice harbouring a *Sin3a* allele that could be deleted conditionally (*Sin3a* ^*Flox/−*^) (Dannenberg et al., 2005). Resulting ES cell lines were then stably transfected with a construct allowing expression of CreER^T2^, in which the Cre recombinase is fused to a modified estrogen receptor (Feil et al., 1997). Addition of 4-hydroxytamoxifen to *Sin3a* ^*Flox/−*^ *:CreER* ^*T2*^ ES cells rapidly induced deletion of the floxed *Sin3a* allele (data not shown) and loss of Sin3a protein (Fig. 5D). As was seen in ICM cells, *Sin3a* ^−/−^ ES cells were not viable and began to die by apoptosis.

Propidium iodide staining and flow cytometry showed a marked accumulation of *Sin3a*-deleted cells with a 4 C DNA content, as compared with heterozygous controls (Fig. 5A). Furthermore, the fraction of 4 C cells entering mitosis, as assessed by the mitotic marker phospho-histone H3 (Ser10), was significantly (p < 0.001) reduced in *Sin3a*-deleted cells (Fig. 5B). Floating and attached cells in tamoxifen-treated and untreated *Sin3a* ^*Flox/−*^ *:CreER* ^*T2*^ cultures were assayed for apoptosis by probing with fluorescently labelled Annexin V, which binds to phosphatidyl serine on the cell membrane of apoptotic cells. By 36 h after tamoxifen addition, approximately 68% of ES cells deleted for *Sin3a* undergo apoptosis, as compared with approximately 12% of untreated cells (Fig. 5C). Taken together, these results indicate that cells lacking Sin3a arrest at the G2/M checkpoint and die by apoptosis.

Given the lack of a functional G1/S damage checkpoint in ES cells, and prior gene expression evidence implicating Sin3a in DSB repair pathways in somatic cells, we hypothesised that the observed G2 arrest in ES cells upon loss of Sin3a could arise from failure to resolve DSBs. Indeed, probing whole-cell lysates from untreated and tamoxifen-treated ES cells revealed a rapid increase in ATM/ATR phosphorylation targets γH2AX (S139), phospho-53BP1 (S166), and phospho-SMC1 (S966), coincident with the loss of Sin3a (Fig. 5D). Whereas H2AX and 53BP1 are hyperphosphorylated in response to a variety of DSB types, SMC1 is typically phosphorylated by ATM as part of the S-phase damage checkpoint (Kim et al., 2002; Yazdi et al., 2002). An increase in the abundance of these markers is consistent with DSBs arising during DNA replication in the absence of Sin3a. From this, we conclude that Sin3a is required to prevent or repair DSBs that arise in ES cells, possibly during DNA replication.

**Fig. 6:**
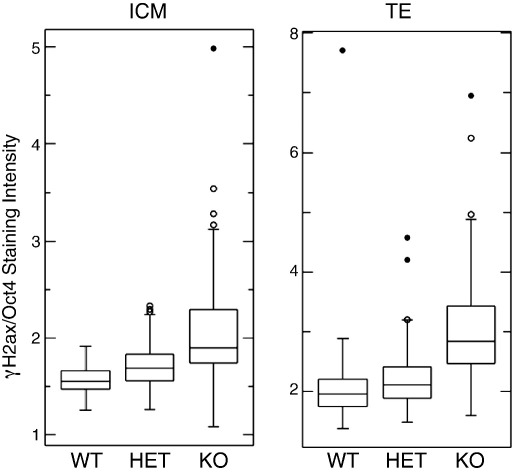
DNA damage is apparent in *Sin3a*^−/−^ ICMs and trophectoderms. Mean intensity values of anti-γH2AX antibody staining relative to anti-Oct4 staining in 3.5 dpc embryos was quantitated and is displayed in box plot format for wild-type (WT), heterozygous (HET) and *Sin3a*-null (KO) blastocysts. Results are shown for cells in the ICM (left hand plot) and in the trophectoderm (TE) based upon Oct4 staining intensity and location within the blastocyst. In both cases the difference between KO and either WT or HET is highly significant (p < 0.0001) according to a single tailed Mann–Whitney test.

To test whether failure to resolve replicative damage could account for the apparent p53-independent apoptosis seen in mutant ICMs, we next quantified levels of γH2AX in *Sin3a* ^−/−^ blastocysts by immunofluorescence. Intensity of anti-γH2AX staining was measured in individual ICM and trophectoderm (TE) nuclei and normalised to anti-Oct4 staining intensity in the same cells. Plotting the resulting distributions reveals that both *Sin3a* ^−/−^ ICM and TE cells indeed have significantly increased H2AX staining as compared to wild-type or heterozygous littermates (Fig. 6 ). It is important to note, however, that increased apoptosis was observed only in ICM cells of *Sin3a* ^−/−^ embryos (Fig. 3), highlighting the distinct repair pathways in pluripotent *vs.* trophectoderm cells. Thus both the *ex vivo* and *in vivo* results are consistent with unresolved DNA damage leading to apoptosis of pluripotent cells in the absence of Sin3a.

### Sin3a associates with cell cycle control and DNA damage response proteins in ES cells

Sin3a has been reported to interact with key cell cycle regulatory proteins such as p53 and Rb, but these proteins play minor roles, if any, in self-renewal of pluripotent cells. To gain a better understanding of how Sin3a may interact with the cell cycle and/or DNA repair machinery in pluripotent cells, we took an unbiased approach to identifying its interacting proteins in ES cells. An epitope-tagged Sin3a protein was expressed in embryonic stem cells and used to pull down the Sin3a complex and any interacting proteins (Fig. S1). The identity of the major interacting proteins was determined by mass spectrometry (Table 2 ).

This strategy identified known components of the Sin3a complex with high peptide numbers and Mascot scores, whereas very few peptides from complex members were obtained from cells expressing the 3xFLAG epitope alone (Table 2). Data were obtained from four independent pull down experiments, providing a list of very high confidence interactors. The related protein Sin3b, which has been reported to interact with Sin3a in NIH3T3 cells (Lienert et al., 2011), was not significantly enriched in the Sin3a immunoprecipitate compared to control cells. This indicates that Sin3a and Sin3b function independently in ES cells, consistent with the dramatic difference in the requirement for Sin3a and Sin3b during embryogenesis [this study and (Cowley et al., 2005; Dannenberg et al., 2005; David et al., 2008)]. A number of known interactors implicated in control of cell cycle and/or proliferation were found co-purifying with Sin3a in ES cells, including Ogt (Hart et al., 2007; Yang et al., 2002), Ing1 (Nikolaev et al., 2004), Mnt (Meroni et al., 2000) and Max (Laherty et al., 1997).

In addition to Sin3a complex components and known interactors, we also identified a number of proteins not previously known to interact with the Sin3a complex. Two of these novel interacting proteins, Bbx and Ppp1cc, have been implicated in cell cycle control (Lee et al., 2010a; Sánchez-Díaz et al., 2001). The interaction with Bbx is particularly robust as it co-purifies with Sin3a with scores and peptide numbers comparable to those seen for bona fide Sin3a complex components. Notably, six of the high confidence (Mascot Scores ≥ 700) interacting proteins are phosphorylated upon DNA damage by ATM or ATR (Matsuoka et al., 2007). Therefore we conclude that Sin3a is intimately linked to regulators of cell cycle and the DNA damage response in ES cells.

### Discussion

Gene regulation by a number of chromatin-modifying complexes has been demonstrated to be important for early murine development, but not necessarily for the survival of pluripotent cells (Niwa, 2007). The Sin3a/HDAC complex is a critical co-repressor of transcription networks that govern cell cycle control, DNA replication and repair, and apoptosis in somatic cells (Cowley et al., 2005; Dannenberg et al., 2005). The requirement for genomic integrity differs considerably between somatic cells and pluripotent cells of the ICM, and correspondingly so does the regulation of these vital processes. Here we show that pluripotent cells exhibit a specific requirement for Sin3a function during early mouse development. Whereas the epiblast compartment is completely lost in Sin3a mutant embryos shortly after implantation, the trophectoderm cells remain largely unaffected. Despite lacking key cell cycle regulatory proteins that interact with Sin3a in somatic cells, Sin3a is required for proper cell cycle control in both undifferentiated ES cells and in embryonic cells fully two days prior the point when they convert to canonical cell cycle regulation (i.e. gastrulation at E6.5). Our data are consistent with Sin3a specifically protecting pluripotent cells from DNA damage leading to cell cycle arrest and elimination by apoptosis both in culture and in implantation stage embryos.

The cell death phenotype we observe in the ICM upon Sin3a loss is likely to be independent of p53, as we did not observe induction of the direct p53 targets *p21* ^*Cip*^, *Apaf1*, and *Dram* upon *Sin3a* deletion. This is consistent with previous reports that deletion or inactivation of p53 in MEFs fails to abrogate the growth and apoptosis phenotypes caused by *Sin3a* deletion (Cowley et al., 2005; Dannenberg et al., 2005) and reports that p53 is largely dispensable for apoptosis both induced by double-stranded breaks in ES cells (Aladjem et al., 1998) and as part of normal development prior to gastrulation (Armstrong et al., 1995; Copp, 1978; El-Shershaby and Hinchliffe, 1974; Macleod et al., 1995). However, the relationship between p53 and DNA damage in pluripotent embryonic cells remains controversial (Corbet et al., 1999; de Vries et al., 2002; Solozobova et al., 2009), and more recent evidence suggests p53 maintains genomic stability in the early embryo by suppressing pluripotency and promoting differentiation to eliminate damaged cells (Lee et al., 2010b; Lin et al., 2005). Our data support non-canonical roles for p53 in ES cells and suggest that parallel or novel apoptotic pathways should be investigated more thoroughly in the early embryo.

ICM cells deleted for *Sin3a* led to a loss of *E2f1* and *Ccn1e* (*Cyclin E*) expression and a dramatic decrease in both *Mcm2* and *Ccn1b* (*Cyclin B*) transcript levels. This increased repression is counter-intuitive, especially when considering that Mcm genes, Cdk/cyclins, and other Myc/E2F targets are *de*-repressed in somatic cells lacking *Sin3a* (Dannenberg et al., 2005). While this decrease in transcription might simply be a consequence of cells whose proliferation has been arrested at G2 by genotoxic stress (see below), the situation is likely more complex in the ICM. In this rapidly proliferating cell type, Myc and E2F targets that drive the cell cycle are very highly expressed during all phases of the cell cycle (Fujii-Yamamoto et al., 2005; Stead et al., 2002), so cells arrested at G2 should still express the factors that drive G1. The notable exception is *Ccn1b* (*Cyclin B1*), which is most highly expressed during G2 in ES cells (Fujii-Yamamoto et al., 2005; Stead et al., 2002) to activate *Cdk1* and promote mitotic entry. However, *Ccnb1* levels are reduced in ICM cells lacking Sin3a (Fig. 4A), the opposite result expected for cells accumulated in G2.

The very high expression levels of Myc and E2F targets in wild-type pluripotent embryonic cells demonstrate that they are not generally repressed by Sin3a as they are in much of the soma. An alternative explanation of our results, therefore, is that Sin3a, also robustly expressed in ES cells and the ICM (Fig. 1A, Fig. 5A), facilitates the high expression of Myc/E2F targets by indirectly activating the expression of Myc and E2Fs themselves through repression of Myc/E2F repressors.

One category of possible Sin3a targets that repress Myc and E2F expression would be miRNAs. Indeed, Myc is known to be downregulated directly by the *let-7* miRNA family (Kumar et al., 2007), whose expression is normally kept very low in ES cells (Marson et al., 2008; Tang et al., 2006a), and *miR-294* is thought to promote Myc activity in ES cells indirectly through repression of an unidentified Myc repressor (Melton et al., 2010). Similarly, human E2f1 is repressed by the *miR-20* miRNA family (O'Donnell et al., 2005), whose members are either nearly or completely undetectable in ES cells and the ICM (Tang et al., 2006a, this study). While we did not observe de-repression of *let-7a*, *miR-20a*, or *miR-17-5p* in ICMs lacking Sin3a, it is possible that other members of these miRNA families, different miRNAs altogether, or traditional protein repressors of Myc or E2f1 are instead misexpressed in these mutant cells, causing loss of Myc/E2F target expression and abortion of the cell cycle.

Our combined observations of severe G2 arrest and phosphorylation of both H2AX and SMC1 upon *Sin3a* deletion suggest a specific role for Sin3a in protecting pluripotent cells against DSBs acquired during replication. However, the cause of this damage might be indirect, especially considering that *S. cerevisiae* lacking Sin3p are hypersensitive to DSBs, but not those specific to S phase (Jazayeri et al., 2004). The reduced Mcm2 levels we observe in *Sin3a*-null ICMs (Fig. 4A) provide a possible explanation. Mammalian cells with reduced Mcm levels are unable to activate a sufficient number of origins to support replication and are prone to stalled forks and double-strand breaks that cause genomic instability and ultimately apoptosis (Bailis et al., 2008; Ekholm-Reed et al., 2004). Because they lack a functional G1 checkpoint, ES cells can readily initiate replication without regard for genome integrity or sufficient available materials to complete the task. Without *Sin3a*, Mcm2 levels in the ICM are considerably reduced, likely from the loss of E2F expression. If these cells nevertheless enter S phase, then the resulting replicative damage from insufficient origins could lead to culling by the functional G2/M damage checkpoint.

It is nevertheless possible that Sin3a plays a more direct role in the response to DNA damage in pluripotent cells. Sin3a directly interacts with p53 and plays an important role in p53-mediated apoptosis in somatic cells (Murphy et al., 1999), but p53 is dispensable in ES cells and in early mouse embryos (Aladjem et al., 1998; Lin et al., 2005; Solozobova et al., 2009). How, then, might the Sin3a complex interact with the cell cycle and/or DNA repair machinery? Our identification of Sin3a interacting proteins in ES cells provides two possible, non-exclusive explanations. Firstly we show that Sin3a in ES cells co-purifies with proteins implicated in cell cycle control. The most abundant of these proteins are Ogt and Bbx. Ogt (O-linked N-acetylglucosamine transferase) is an abundant, well-conserved protein implicated in a number of different cellular processes and required for viability of embryonic stem cells (Hart et al., 2007; Shafi et al., 2000). Ogt has been shown to interact with Sin3a in HepG2 cells and the two proteins were shown to act cooperatively in transcriptional repression (Yang et al., 2002). Bbx, which also co-purifies with Sin3a with similar abundance as bona fide Sin3a complex components (Table 2), is a relatively uncharacterised HMG box-containing protein shown to complement a cell cycle defect in *S. pombe* (Sánchez-Díaz et al., 2001). Its function in mammalian cells remains unknown. Secondly, our analysis identified several Sin3a interacting proteins that become phosphorylated by ATM and/or ATR in response to DNA damage (Table 2), providing a possible direct link between Sin3a and DNA damage response pathways.

In conclusion, we have found that Sin3a is absolutely required for the proliferation and survival of pluripotent embryonic cells around the time of implantation. We propose that Sin3a is crucial for regulating the pluripotent cell cycle by indirectly facilitating Myc and E2F activity in the ICM. Loss of Myc/E2F target expression in *Sin3a*-nulls causes reduced proliferation and errors in DNA replication, activating the stringent G2/M checkpoint that results in death of mutant cells. This is in stark contrast to the situation in T cells or cells of the epidermis, where Sin3a is not required for cellular proliferation (Cowley et al., 2005; Nascimento et al., 2011), and provides insight into the molecular control of both the unusual cell cycle of pluripotent embryonic cells and their genomic integrity.

The following are the supplementary materials related to this article.

Supplementary Fig. 1 Representative immunoprecipitation samples analysed by mass spectroscopy. Colloidal Coomassie blue staining of PAGE lanes containing eluates from anti-FLAG immunoprecipitations from E14tg2a ES cells expressing either Sin3a C-terminally tagged with 3xFLAG (Sin3a 3xFLAG) or 3xFLAG alone (Vector). Lane M is a protein size marker, with molecular weights given in kDa. The two sample lanes pictured were run on separate gels treated identically. Indicated proteins are suggestions based upon molecular weight and abundance, however protein identities were not determined for individual bands.

Supplementary Table 1 Primers.

Supplementary Table 2 Complete mass spectrometry data for Sin3a and control cell lines.

## Figures and Tables

**Table 1:** Genotypes of pups and embryos produced by *Sin3a* heterozygote intercrosses at the indicated stages of embryonic development or after weaning (3 weeks of age).

	+/+	+/−	−/−	N
3.5 d.p.c	28 (28.6%)	49 (50.0%)	21 (21.4%)	98
4.5 d.p.c	25 (31.6%)	40 (50.6%)	14 (17.7%)	79
5.5 d.p.c	21 (32.8%)	39 (60.9%)	4 (6.3%)	64
Weaning	28 (35%)	52 (65%)	0 (0%)	80

**Table 2:** Highest-scoring Sin3a interactors in ES cells.

Protein name	Max mascot score^a^	Unique 576^b^	Total 576^c^	Unique 590^b^	Total 590^c^	Unique 663E4^b^	Total 663E4^c^	Unique 663E5^b^	Total 663E5^c^	Control Unique^b^	Control Total^c^	Entrez ID	Comment
Sin3a	5086	64	458	59	537	55	717	58	605	0	0	20466	Sin3a complex component
Hdac1	2118	26	194	19	260	19	263	23	269	8	12	433759	Sin3a complex component
Arid4b	1867	25	75	18	64	29	107	19	76	3	3	94246	Sin3a complex component
Sap130	1849	21	140	23	166	22	216	23	185	4	4	269003	Sin3a complex component
Arid4a	1609	0	0	0	0	24	109	24	85	0	0	238247	Sin3a complex component
Rbbp7	1474	15	112	14	142	13	196	16	227	1	1	245688	Sin3a complex component
Hdac2	1341	19	141	13	116	17	155	20	159	0	0	15182	Sin3a complex component
Suds3	1309	18	124	15	119	16	177	15	156	0	0	71954	Sin3a complex component
Brms1l	1220	16	60	13	76	12	66	12	58	0	0	52592	Sin3a complex component
Rbbp4	1136	13	83	12	125	12	127	11	143	5	5	19646	Sin3a complex component
Ing2	1013	11	26	6	20	4	38	6	26	0	0	69260	Sin3a complex component
Sap30	892	13	53	6	53	9	60	7	46	0	0	60406	Sin3a complex component
Brms1	855	10	35	10	47	12	66	12	58	0	0	107392	Sin3a complex component
Sap30l	629	8	16	6	13	7	35	6	32	0	0	50724	Sin3a complex component
Sap25	316	5	11	3	3	1	2	1	2	0	0	751865	Sin3a complex component
Ogt	3371	43	84	33	110	27	123	27	80	1	1	108155	Known interactor: (Yang et al., 2002)
Bbx	1837	26	59	19	78	19	120	22	92	0	0	70508	HMG box transcription factor that is necessary for cell cycle progression from G1 to S phase (Sánchez-Díaz et al., 2001)
Tet1	1699	0	0	0	0	24	111	29	96	0	0	52463	Known interactor: (Williams et al., 2011)
Foxk2	1359	18	19	12	26	16	21	10	19	0	0	68837	Phosphorylated upon DNA damage, probably by ATM or ATR: (Matsuoka et al., 2007)
Pspc1	1055	13	23	11	23	13	20	9	16	2	2	66645	Phosphorylated upon DNA damage, probably by ATM or ATR: (Matsuoka et al., 2007)
Fam60a	901	11	34	12	53	8	49	11	52	0	0	56306	Phosphorylated upon DNA damage, probably by ATM or ATR: (Matsuoka et al., 2007)
Phf23	765	6	16	9	25	4	21	5	21	0	0	78246	Phosphorylated upon DNA damage, probably by ATM or ATR: (Matsuoka et al., 2007)
Bahcc1	726	7	14	10	16	4	5	7	11	2	2	268515	Phosphorylated upon DNA damage, probably by ATM or ATR: (Matsuoka et al., 2007)
Foxk1	702	12	9	6	14	7	7	15	15	0	0	17425	Phosphorylated upon DNA damage, probably by ATM or ATR: (Matsuoka et al., 2007)
2810046L04Rik	551	10	14	3	5	4	7	4	6	0	0	212127	Uncharacterised protein KIAA2032 homolog
Cbx3	499	2	10	6	2	0	0	0	0	2	2	12417	Hp1gamma
Zglp1	453	5	6	2	2	1	2	1	3	0	0	100009600	GATA-like protein 1
Ing1	428	8	13	2	18	4	38	2	23	0	0	26356	Known interactor: (Nikolaev et al., 2004).
Ppp1cc	369	6	5	4	7	0	0	5	5	0	0	19047	Implicated in cell cycle progression during the transition from mitosis into interphase: (Lee et al., 2010a)
Dnajb6	346	5	6	2	2	1	2	1	2	0	0	23950	DnaJ homolog subfamily B member 6
Mnt	332	5	5	1	1	0	0	3	3	0	0	17428	Known interactor: (Meroni et al., 2000)
Ythdf1	284	3	4	1	1	1	1	0	0	0	0	228994	YTH domain family protein 1
Hbp1	263	4	6	3	4	0	0	4	7	0	0	73389	Known interactor: (Swanson et al., 2004)
Klf13	242	2	2	3	3	2	2	1	1	0	0	50794	Known interactor: (Kaczynski et al., 2001)
Tet2	226	5	6	4	5	1	1	0	0	0	0	214133	Methylcytosine dioxygenase
H2afj	235	3	10	3	14	3	8	0	0	4	12	232440	Histone H2A.J
Hist2h2ac	235	3	10	3	14	3	8	0	0	0	0	8338	Histone H2A type 2-C
Max	188	2	3	1	1	2	2	1	1	0	0	17187	Known interactor: (Laherty et al., 1997)

a Maximum mascot score from the four different experiments.

b Number of unique peptides obtained from individual experiments.

c Total number of peptides obtained from individual experiments.
