# Supplementary material for: Sin3a is essential for the genome integrity and viability of pluripotent cells
Source: Dev Biol. 2012 Mar 1;363-318(1-12):62–73. doi: 10.1016/j.ydbio.2011.12.019 (PMC3334623; doi:10.1016/j.ydbio.2011.12.019)
Supplement: Supplementary Fig. 1 — Representative immunoprecipitation samples analysed by mass spectroscopy. Colloidal Coomassie blue staining of PAGE lanes containing eluates from anti-FLAG immunoprecipitations from E14tg2a ES cells expressing either Sin3a C-terminally tagged with 3xFLAG (Sin3a 3xFLAG) or 3xFLAG alone (Vector). Lane M is a protein size marker, with molecular weights given in kDa. The two sample lanes pictured were run on separate gels treated identically. Indicated proteins are suggestions based upon molecular weight and abundance, however protein identities were not determined for individual bands. [file mmc1.pdf]

Supplementary Table 1. Primers

qPCR Primers for Single ICM expression studies

Gene	Forward (5’–3’)	Reverse (5’–3’)
Sin3a	TTCCCTCTTCTGGTTCTCGGCCTC	GCTGGTGGATGGCACTAGGGC
Oct4	GGAAAGCAACTCAGAGGGAA	TTCTAGCTCCTTCTGCAGGG
Nanog	ATGCCTGCAGTTTTTCATCC	GAGGCAGGTCTTCAGAGGAA
E2f1	GGATGGGCTGGAGAAACAGTC	CCATAGGAAGGACGCATACCC
Cyclin E1	CCAGCAGTAAGAAGGAGCAGC	CCTCTCCATAGAGCACAGCATC
Cyclin B1	TGCCCTCCACAGTGTTCTTAAATG	GTGGCATTACAAGACAGGAGTGG
Mcm2	GAAGGATCTGATGGACAAGGCC	GGAAGTGGGAAGTGACGTAGG
$p21^{Cip}$	AAAGTGTGCCGTTGTCTCTTCG	AGTCAAAGTTCCACCGTTCTCG
Apaf1	GGCTCATCTGTTCTGTAGCTGG	GTGTGAGCCACAGCCATCTTC
Dram	ATTGTGTGACAACTCTCTCCTGG	TCTCGGGTACAAAGTCCAAGG

Gene-specific forward primers for amplification of miRNA cDNAs

miRNA	Forward Primer (5’–3’)
miR-16	ACACTCCAGCTGGGTAGCAGCACGTAAATA
miR-17-5p	ACACTCCAGCTGGGCAAAGTGCTTACAGTGCA
miR-20a	ACACTCCAGCTGGGTAAAGTGCTTATAGTGC
miR-291-3p	ACACTCCAGCTGGGAAAGTGCTTCCACTTTG
miR-292-3p	ACACTCCAGCTGGGAAGTGCCGCCAGGTTTT
miR-293	ACACTCCAGCTGGGAGTGCCGCAGAGTTTG
miR-294	ACACTCCAGCTGGGAAAGTGCTTCCCTTTT
miR-295	ACACTCCAGCTGGGAAAGTGCTACTACTTTT
let-7a	ACACTCCAGCTGGGTGAGGTAGTAGGTTG
