# Supplementary figures and images for: Sin3a is essential for the genome integrity and viability of pluripotent cells
Source: Dev Biol. 2012 Mar 1;363-318(1-12):62–73. doi: 10.1016/j.ydbio.2011.12.019 (PMC3334623; doi:10.1016/j.ydbio.2011.12.019)

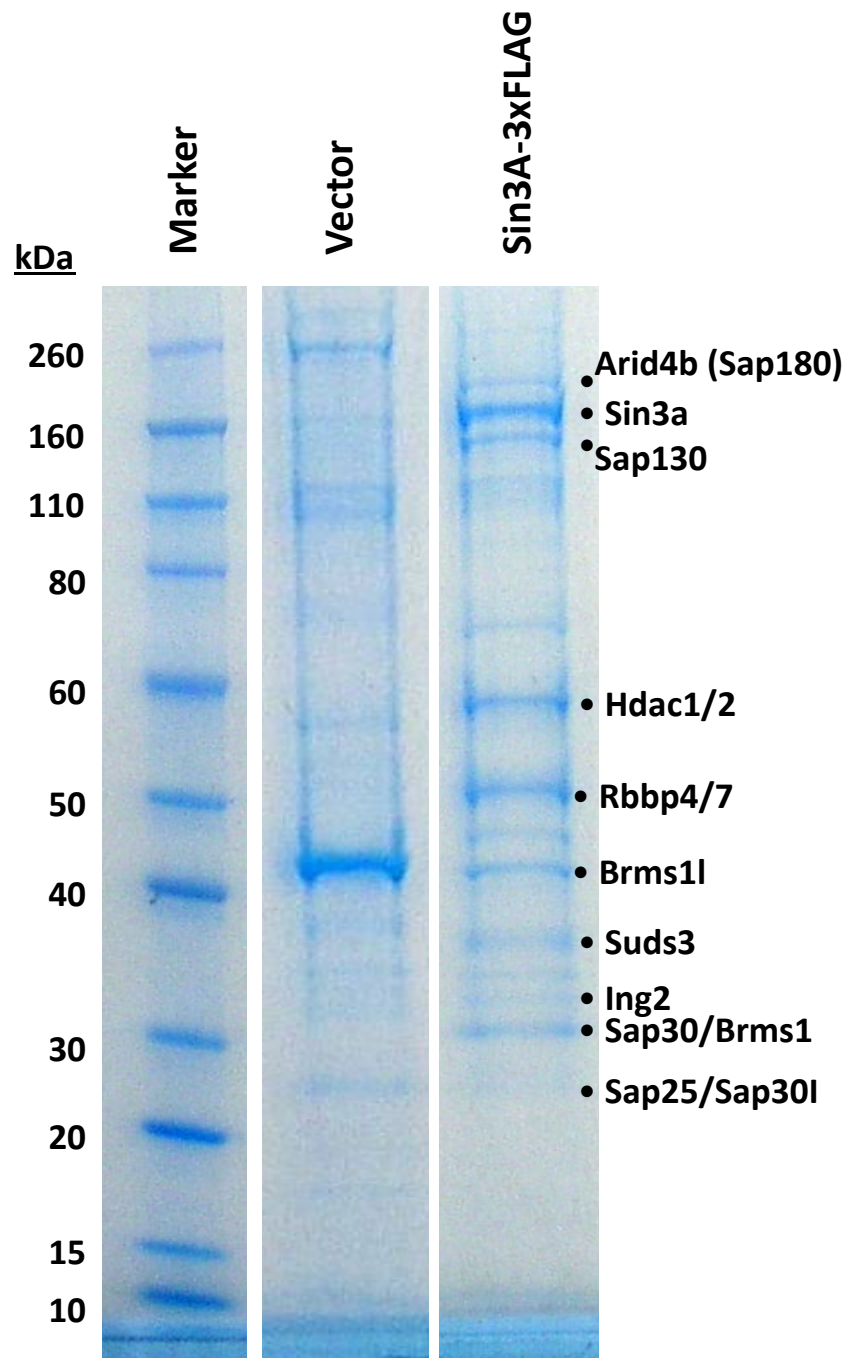

Supplement: Supplementary Table 1 — Primers. [file mmc2.pdf]
